# Successful Management of Generalized Drug-Influenced Gingival Enlargement: A Clinical Case Report

**DOI:** 10.7759/cureus.116089

**Published:** 2026-09-11

**Authors:** Reshama Bansal, Neha D Patle, Madhu S Ratre, Shaleen Khetarpal

**Affiliations:** 1 Periodontology, Government College of Dentistry, Indore, Indore, IND

**Keywords:** anticonvulsants, gingival overgrowth, gingivectomy, periodontal therapy, phenytoin

## Abstract

Drug-influenced gingival enlargement (DIGE) is a clinically significant adverse effect of medications such as phenytoin and may be exacerbated by plaque-induced inflammation. The interaction between drug-related alterations in gingival connective-tissue metabolism and local inflammatory factors can result in marked gingival enlargement, compromising oral hygiene and periodontal health. We present a case of a 40-year-old man with a long history of phenytoin use who developed generalized, bulbous, and fibrotic gingival enlargement associated with periodontal pocketing. The management of DIGE includes plaque control and, when clinically feasible, the modification or substitution of the offending medication in consultation with the treating physician. Following initial nonsurgical periodontal therapy, local inflammation improved; however, residual fibrotic gingival enlargement warranted surgical intervention. External bevel gingivectomy was therefore performed to remove the excessive tissue, reduce pockets to maintainable levels, and establish a physiologic and maintainable gingival contour. Histopathological examination confirmed fibroepithelial hyperplasia. Following surgery, periodontal pockets were markedly reduced, with no furcation involvement. This case is clinically relevant because it demonstrates the successful long-term management of phenytoin-associated gingival enlargement despite the continuation of the offending medication, with satisfactory periodontal maintenance and no recurrence over three years of follow-up, highlighting the potential for sustained periodontal stability with appropriate surgical management and maintenance.

## Introduction

Drug-influenced gingival enlargement (DIGE) is a well-documented adverse effect associated with the long-term use of certain systemic medications, most commonly anticonvulsants, immunosuppressants, and calcium channel blockers. Among these, phenytoin, a hydantoin derivative widely prescribed for the management of epileptic disorders, is one of the earliest and most frequently implicated drugs in the development of gingival overgrowth [1]. Phenytoin-influenced gingival enlargement (PIGE) typically presents as a generalized fibrotic enlargement of the gingival tissues, often beginning at the interdental papillae and gradually extending to involve marginal and attached gingiva [1,2].

Phenytoin showed the greatest incidence of gingival hyperplasia among the anticonvulsants evaluated, ranging from 15.61% to 73% [2]. The pathogenesis involves heterogeneity among gingival fibroblast populations, altered collagen turnover, inflammatory mediators, growth factors, and interactions between drug exposure and plaque-induced inflammation [3-5].

Clinically, PIGE can result in aesthetic concerns and disfigurement, impaired mastication and speech, difficulty in maintaining oral hygiene, and an increased risk of other dental infections associated with pain and discomfort. If left untreated, the condition can significantly compromise periodontal health and negatively impact the patient's quality of life. The severity of gingival enlargement does not always correlate directly with serum phenytoin levels, highlighting the role of individual susceptibility and local inflammatory factors [6].

This case report presents a case of generalized DIGE associated with long-term phenytoin therapy, highlighting its clinical features, diagnosis, and management. The case is particularly noteworthy for the absence of recurrence over three years despite the continuation of the offending medication, demonstrating that sustained periodontal stability may be achieved through appropriate surgical management, meticulous plaque control, and long-term periodontal maintenance when the modification of the causative medication is not clinically feasible.

## Case presentation

A 40-year-old male patient presented to the department of periodontology with a chief complaint of generalized gingival enlargement involving the maxillary and mandibular arches and difficulty in closing his lips for the past few years. He reported difficulty in maintaining oral hygiene, mastication, and closing his lips. He also reported pus discharge from the gums over the past few months. Medical history revealed that the patient had been on antiepileptic medication (Eptoin {phenytoin sodium 100 mg} and Torvate {sodium valproate 500 mg}) twice daily for the past 20 years. There has been no episode of seizure for the past 15 years. Personal history revealed that the patient had been a tobacco chewer for 15 years and had discontinued the habit 3-4 months before presentation. The patient had previously sought care at local healthcare facilities but did not receive definitive treatment.

Clinical examination

Extraoral examination revealed difficulty in complete lip closure due to the extensive gingival enlargement, resulting in a disfiguring appearance of the lower third of the face. The patient was neurologically intact and well-oriented and did not show any signs of deficit, such as visual impairment, difficulty in swallowing and speaking, confusion, and disorientation to time and place. Vital signs were within normal limits.

Intraoral examination revealed grade III generalized bead-like gingival enlargement (according to the Angelopoulos and Goaz index), as the enlargement was covering more than two-thirds of the crown portion, involving the interdental papillae, marginal gingiva, and attached gingiva (Figure 1A-1E) [7].

**Figure 1:**
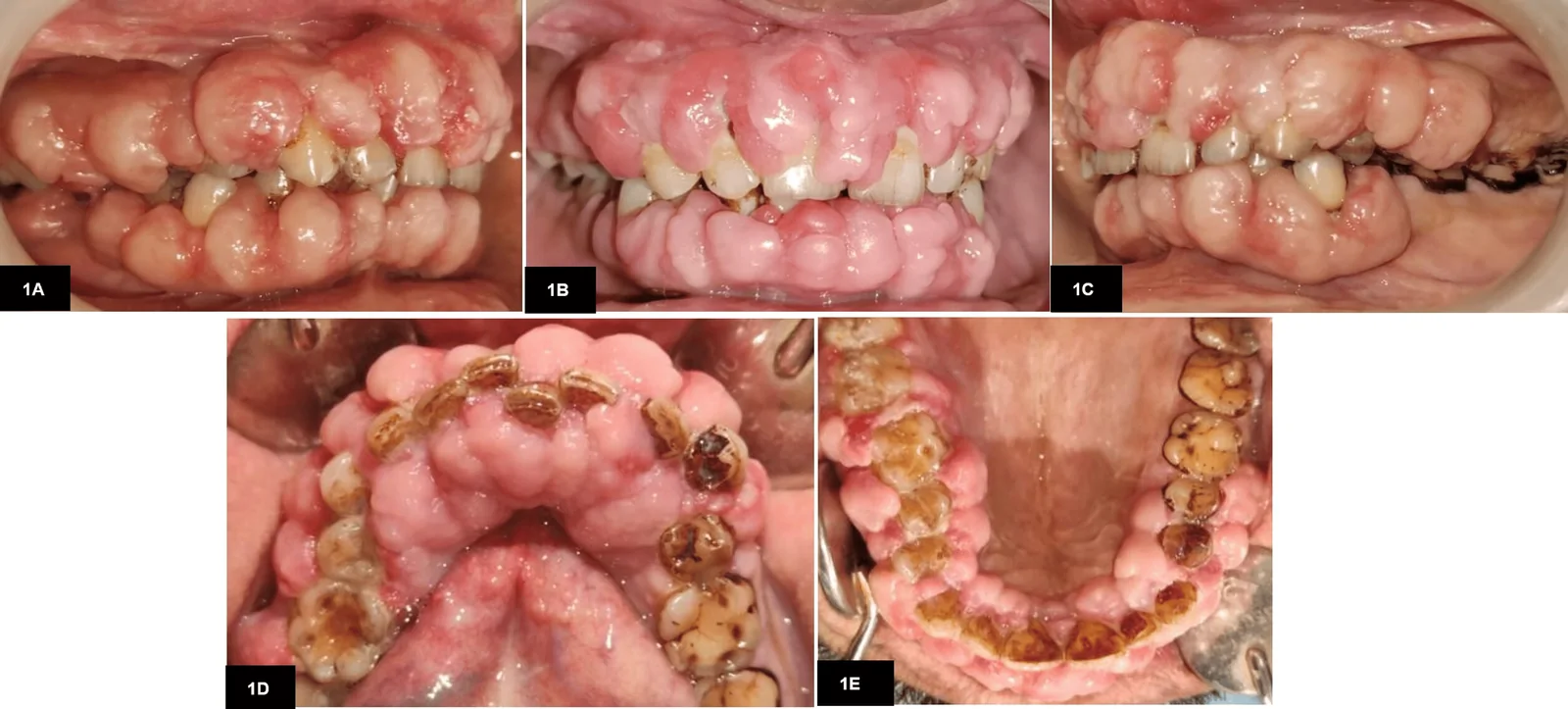
Clinical presentation at first visit

The enlargement extended to the occlusal/incisal third of the crowns of all teeth. The enlarged gingiva exhibited a lobulated appearance with deep cleft-like grooves separating adjacent lobules. The gingiva was predominantly pink in color with focal erythematous areas and was firm and fibrotic in consistency, with superimposed inflammatory enlargement. Periodontal examination revealed generalized bleeding on probing and purulent discharge, with no tenderness on palpation. Periodontal examination revealed 32 teeth present, with generalized combined periodontal pockets, probing pocket depths of ≥10 mm at affected sites, and clinical attachment levels of 4-5 mm. Bleeding on probing was present at approximately 80% of the examined sites. No furcation involvement was detected. Local contributing factors included abundant plaque, heavy supragingival and subgingival calculus deposits, and extensive extrinsic tobacco staining. The generalized tooth mobility of varying grades was also observed.

Radiographic evaluation revealed generalized horizontal alveolar bone loss, which was mild in the mandibular posterior region and moderate to severe in the remaining dentition, with angular bone loss with teeth number 16 and number 28 (Figure 2).

**Figure 2:**
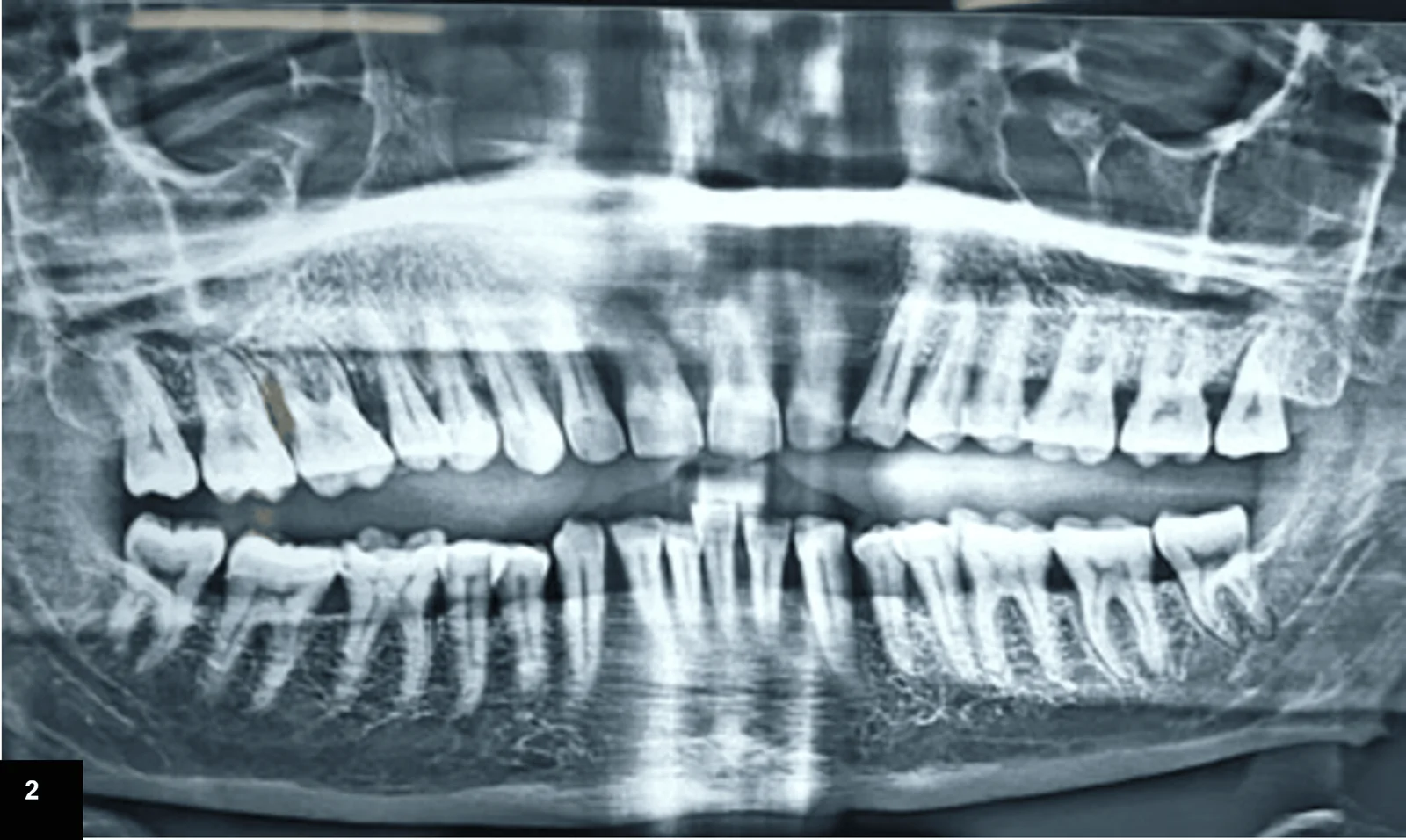
Preoperative radiograph

Tooth number 11 demonstrated bone loss extending to the middle third of the root, which correlated clinically with grade II mobility.

All hematological investigations were within normal limits, including complete blood count (CBC), bleeding time (BT), clotting time (CT), random blood sugar (RBS), HIV, and hepatitis B surface antigen (HBsAg).

Based on the patient's medical history and clinical findings, a provisional diagnosis of localized stage III grade C periodontitis and severe drug-influenced gingival enlargement was made.

Following consultation with the patient's physician, the alteration or substitution of the antiepileptic medication was deemed inappropriate, considering the patient's long-term therapy, satisfactory seizure control, and the potential risks associated with changing the existing regimen. Therefore, a treatment plan consisting of initial nonsurgical periodontal therapy, followed by an external bevel gingivectomy using a scalpel with adjunctive electrocautery, was formulated.

Management

Initial scaling and root planing (SRP) was performed with utmost care to avoid excessive tissue trauma and bleeding from the enlarged gingival tissues, along with thorough patient counseling regarding oral hygiene practices. The patient was prescribed 0.2% chlorhexidine mouthwash twice daily post-SRP. SRP was completed over two to three appointments on a weekly basis until the inflammatory status subsided and bleeding on probing reduced.

Following satisfactory re-evaluation after phase I periodontal therapy (Figure 3), the surgical excision of the gingival enlargement using external bevel gingivectomy with a scalpel was planned to remove the excess bulbous, fibrous tissue and achieve maintainable gingival contour. As the residual periodontal pockets were predominantly suprabony and the angular defects associated with teeth number 16 and number 28 were not considered suitable for regenerative therapy, external bevel gingivectomy was selected as the surgical approach. The procedure was supplemented with electrocautery to achieve adequate hemostasis and optimal gingival contouring. Informed consent was obtained from the patient, along with medical clearance from a general physician, before the procedure. The surgical procedure was planned in a segmental approach to ensure better patient compliance and comfort, hemostasis, and optimal healing.

**Figure 3:**
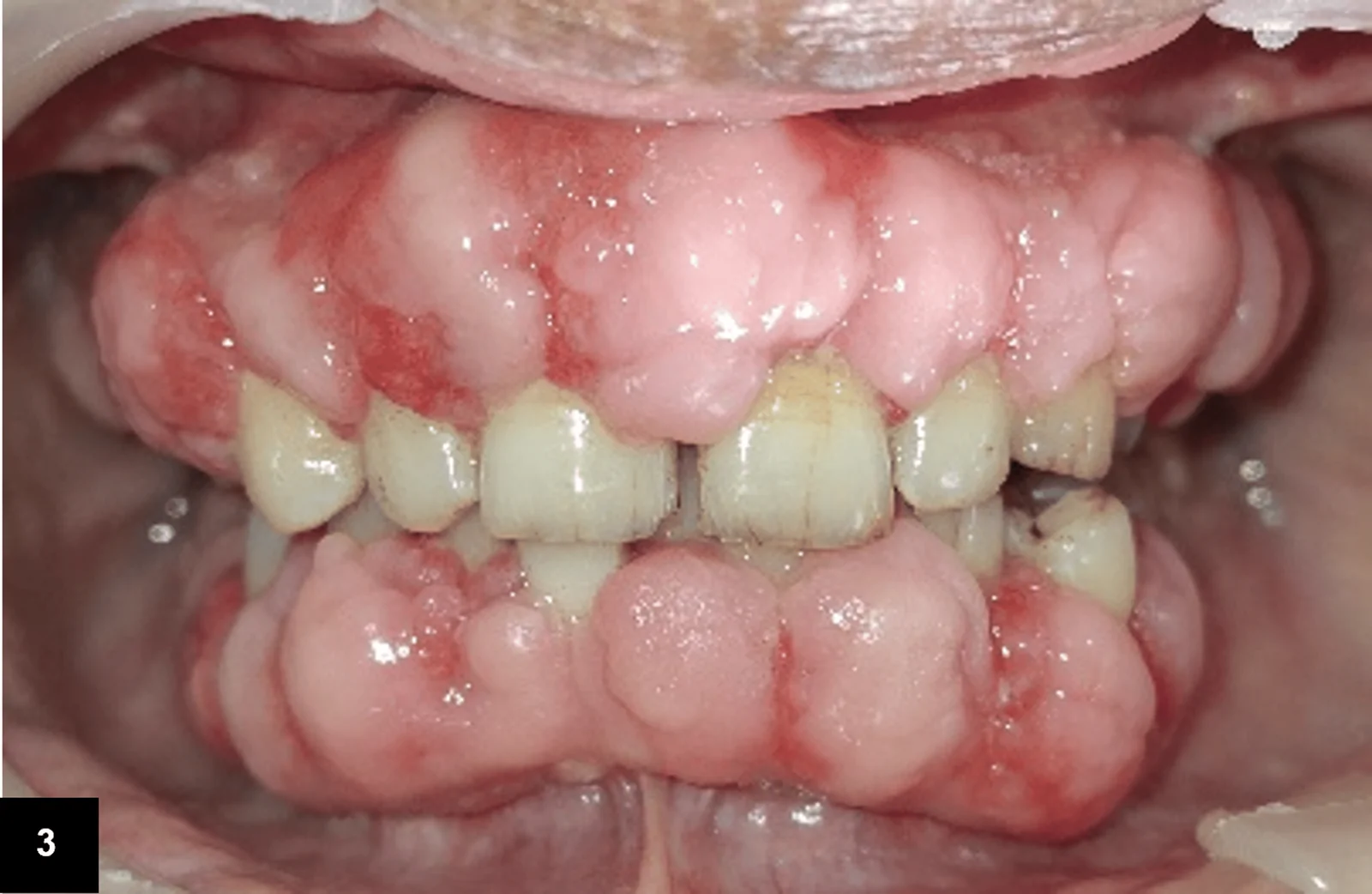
Clinical presentation after phase I therapy

Surgical procedure

Following the administration of local anesthesia, residual periodontal pockets were assessed, and bleeding points were marked using a Crane-Kaplan pocket marker (Figure 4).

**Figure 4:**
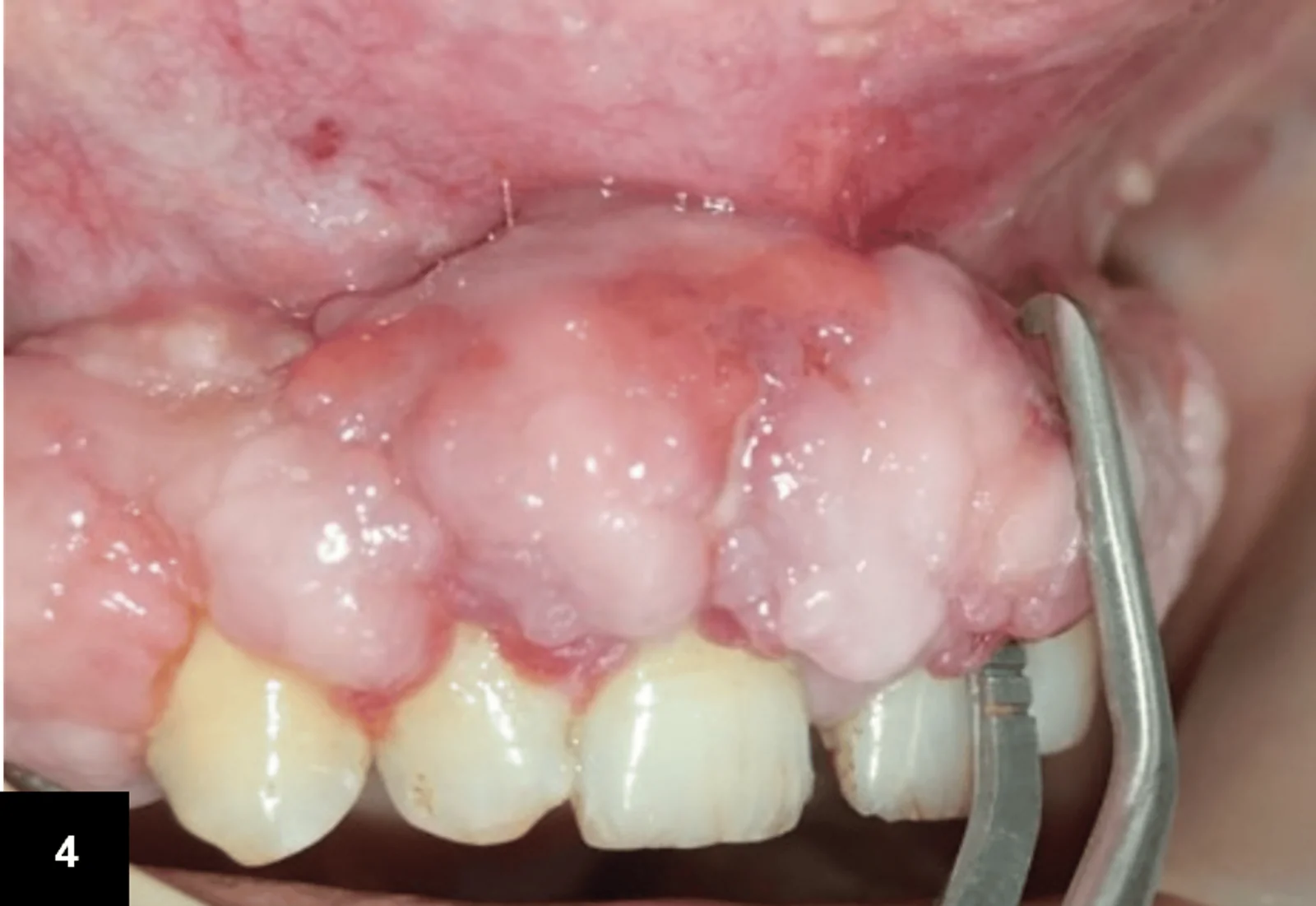
Pocket marking using the Crane-Kaplan pocket marker

External bevel incisions were made using a number 15 blade, maintaining the bevel at approximately 45° to the tooth surface (Figure 5).

**Figure 5:**
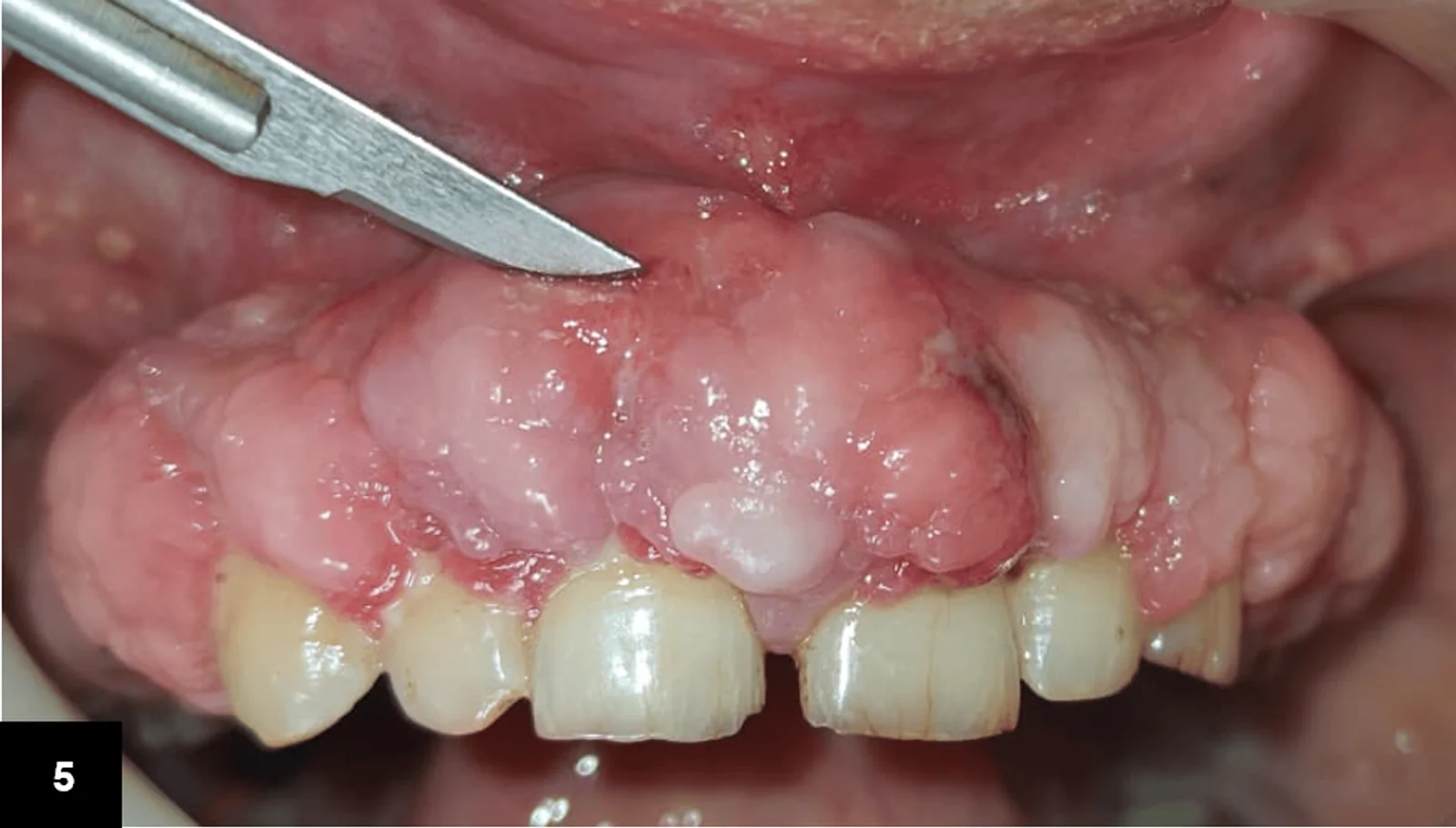
External bevel incision

Once the incisions were completed, the excised tissue was removed using a curette, and all granulation tissue was thoroughly debrided (Figure 6A, 6B).

**Figure 6:**
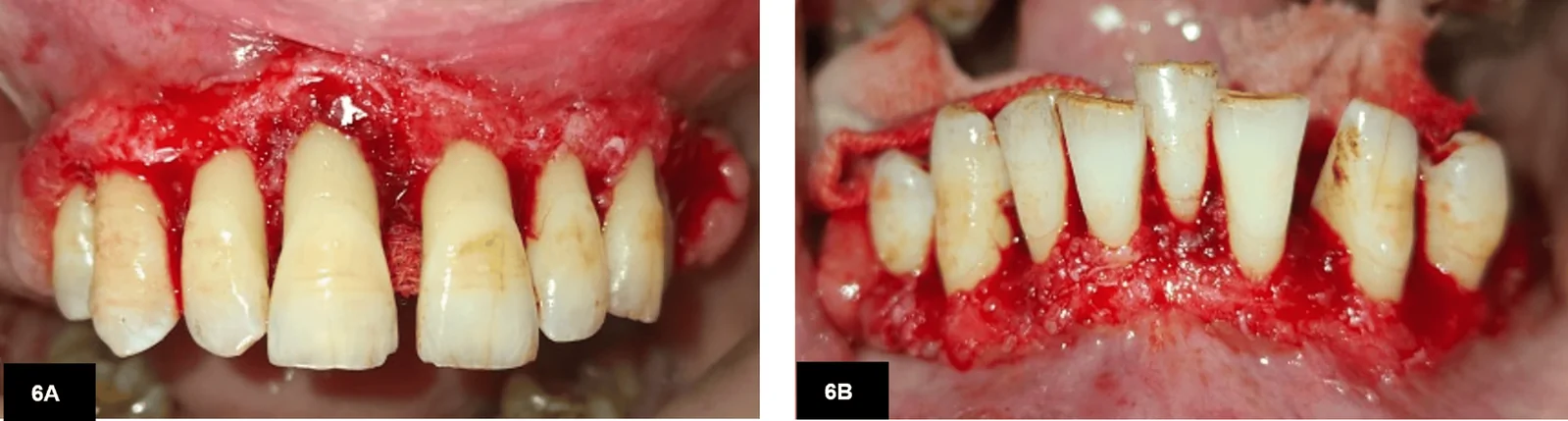
Intraoperative after the excision of the growth

The underlying subgingival calculus was then meticulously removed by scaling, followed by thorough root planing to achieve a clean and biologically compatible root surface. Electrocautery was subsequently used for hemostasis and gingivoplasty (Figure 7A-7E).

**Figure 7:**
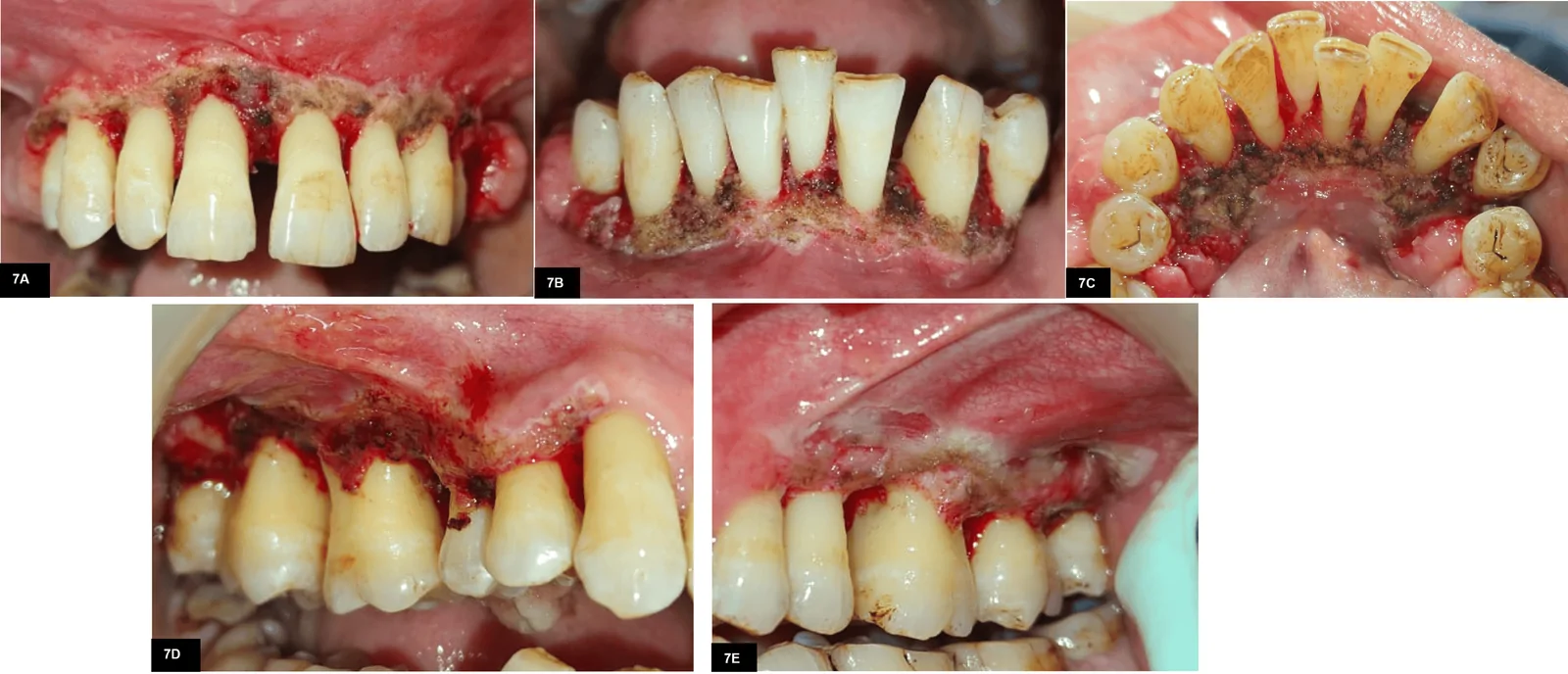
After electrocautery immediate postoperatively

A periodontal dressing (Coe-Pak, GC Corporation, Tokyo, Japan) was placed, and appropriate postoperative instructions were given, and ibuprofen 400 mg was prescribed three times daily for pain management, along with vitamin B and vitamin C supplementation to support postoperative healing (Figure 8).

**Figure 8:**
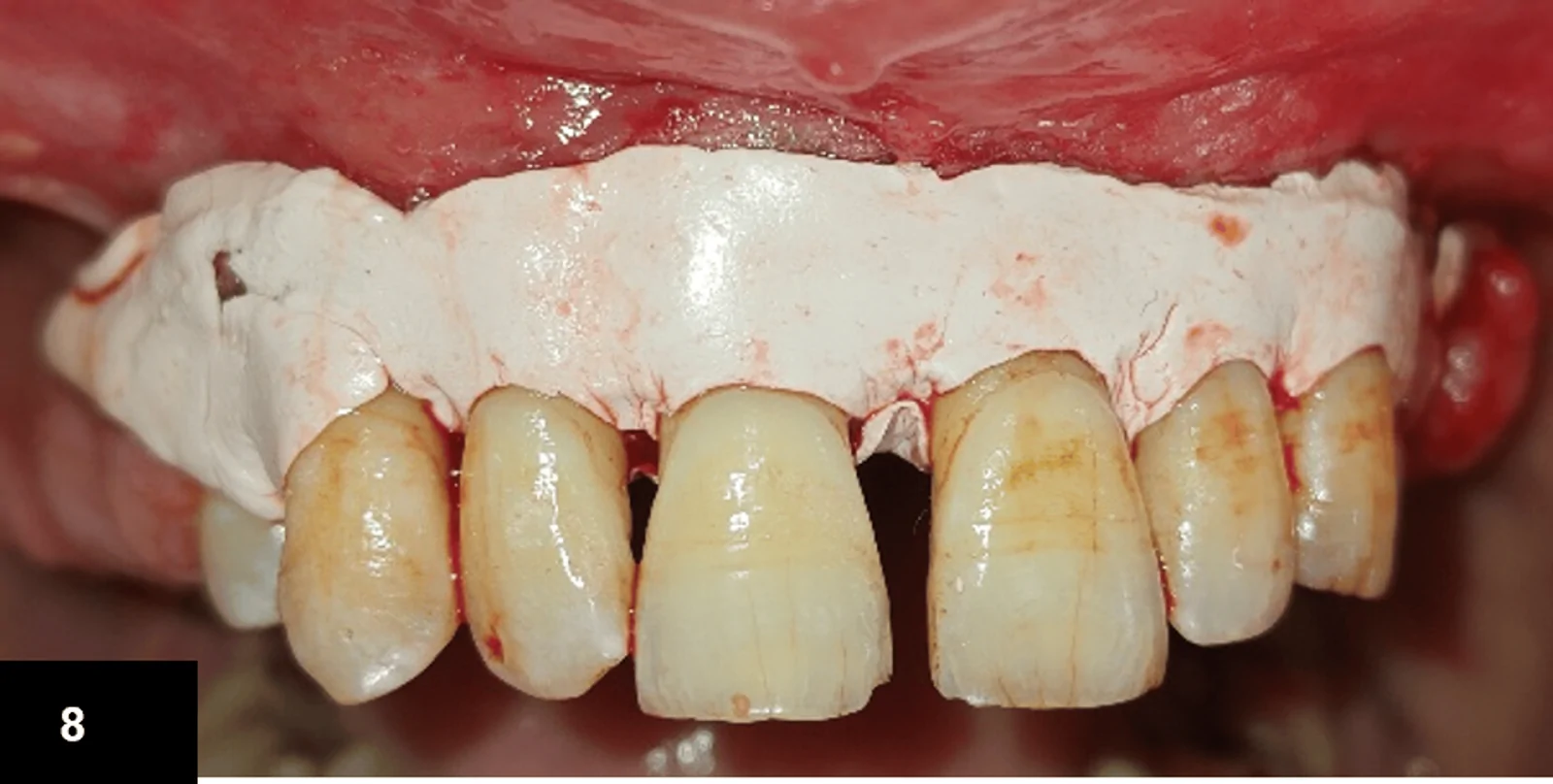
Periodontal dressing given

The patient was recalled after one week for evaluation. The gingivectomy procedure was performed segment-wise at 15-21-day intervals. The excised tissue was sent for histopathological examination.

The patient was recalled monthly for the first six months for professional plaque control and reinforcement of oral hygiene measures, followed by three-monthly maintenance visits for the subsequent year and six-monthly follow-up thereafter for a total period of three years. Re-evaluation during the recall visits demonstrated satisfactory healing, improved gingival contours, and a reduction in the mobility of tooth number 11 from grade II to grade I (Figure 9A, 9B).

**Figure 9:**
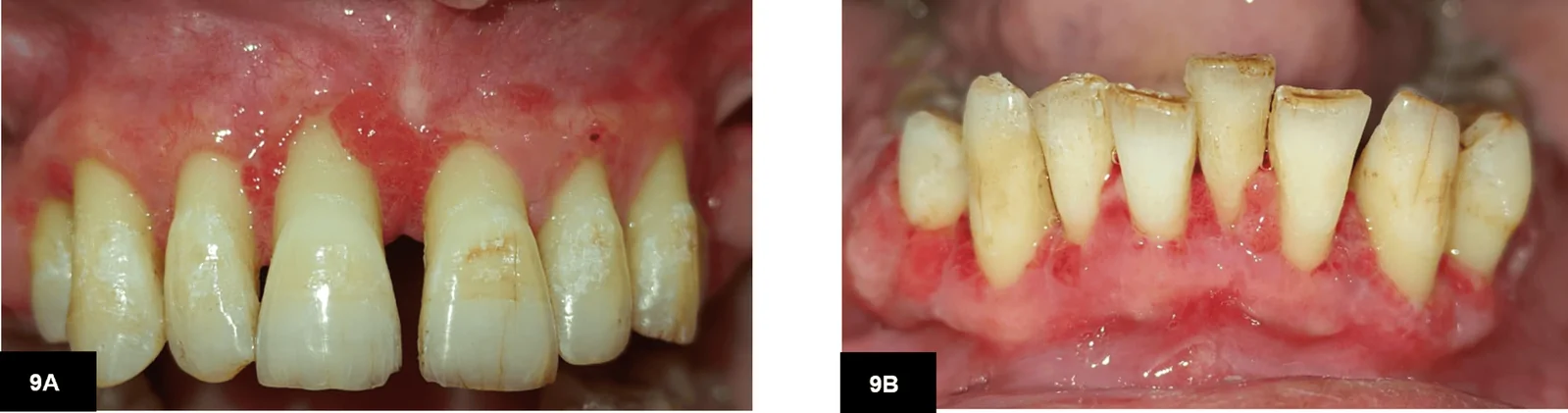
Follow-up after one month

No recurrence was observed during the three-year follow-up period (Figure 10A-10C).

**Figure 10:**
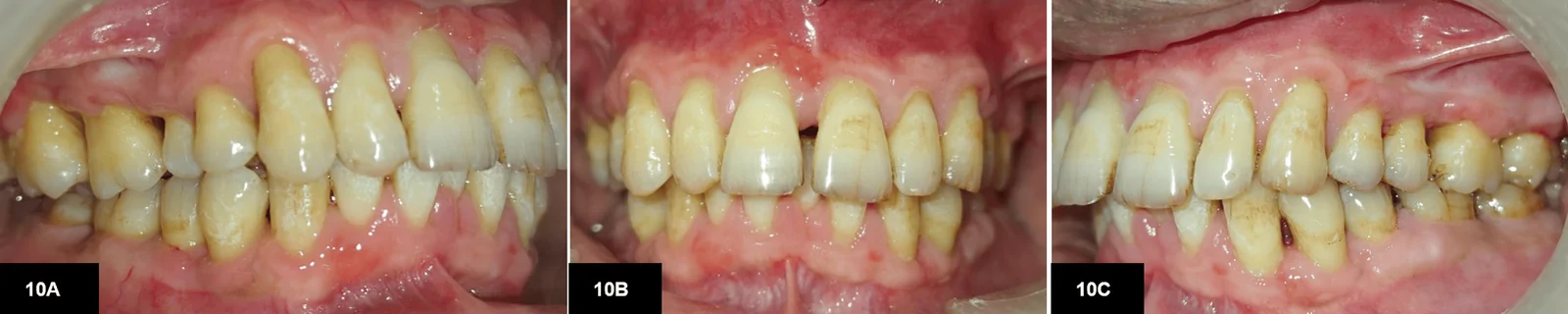
Three-year follow-up

Histopathology

The histological section showed proliferative hyperplastic stratified squamous epithelium with elongated rete ridges. The underlying connective tissue was fibrocellular in nature, with proliferating blood vessels and a marked chronic inflammatory infiltrate comprising lymphocytes and plasma cells. Overall, the histopathological features were suggestive of fibroepithelial hyperplasia (Figure 11).

**Figure 11:**
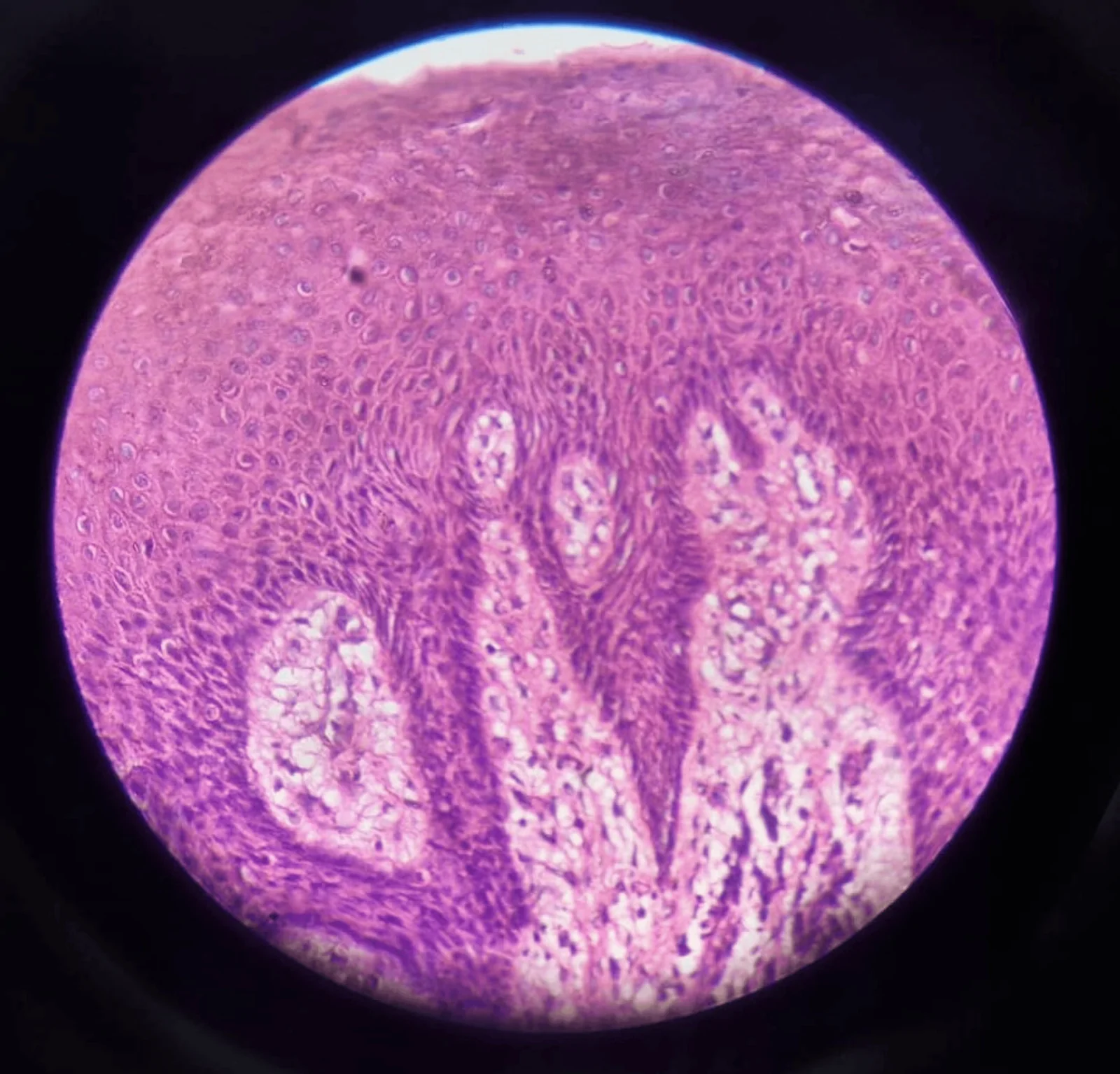
Histopathological section showing epithelial hyperplasia with the prominent elongation of rete ridges, suggestive of proliferative changes

## Discussion

DIGE is a well-recognized adverse effect associated with the long-term use of certain systemic medications, particularly anticonvulsants such as phenytoin [1-4,8]. Since its first description by Kimball in 1939, PIGE has remained a significant clinical problem due to its high prevalence and impact on oral health and the quality of life [9]. The current case demonstrates the clinical presentation and successful management of generalized gingival enlargement associated with chronic phenytoin therapy.

PIGE usually develops within the first few months of drug administration and gradually increases in severity with continued use. Clinical manifestations may become evident as early as one month after the initiation of therapy and may progress over time, often reaching maximal severity within 12-18 months [1,6,8,10]. In the present case, the patient had been receiving phenytoin therapy for approximately two decades. Poor oral hygiene, together with tobacco and areca nut chewing, may have further contributed to the extensive and generalized enlargement observed clinically.

Clinically, the enlargement typically begins as a bead-like enlargement of the interdental papillae and gradually progresses to involve the marginal and attached gingiva. In severe cases, it may coalesce, covering a significant portion of the clinical crowns and resulting in functional and aesthetic concerns. The fibrotic, firm consistency observed clinically reflects the increased collagen content characteristic of PIGE [6].

Only a subset of gingival fibroblasts appears to be susceptible to phenytoin, suggesting that genetic predisposition and differential fibroblast responses are important determinants of the development, severity, and individual variation in the clinical expression of gingival enlargement [11-13]. As the gingival enlargement progresses, plaque-retentive areas are created that further compromise oral hygiene, establishing a self-perpetuating cycle of plaque accumulation, inflammation, and gingival overgrowth.

The management of PIGE requires a comprehensive and multidisciplinary approach. Clinical guidelines suggest that the management of DIGE should initially focus on meticulous plaque control and, when feasible, the modification of the causative medication, with surgical intervention reserved for persistent or severe cases [14]. Surgical intervention is indicated in cases where the enlargement is severe, fibrotic, and unresponsive to nonsurgical therapy [5]. Procedures such as gingivectomy or periodontal flap surgery are performed to restore normal gingival contours, improve function, and enhance aesthetics.

In the present case, combined periodontal pockets were present, and the gingival enlargement exhibited an extensive bulbous, fibrotic morphology. Therefore, external bevel gingivectomy was considered the most appropriate surgical approach, as it allowed the effective removal of the excess fibrotic gingival tissue and the reduction of the pockets to maintainable levels while facilitating the establishment of a physiologic postoperative gingival contour. The surgical intervention resulted in significant clinical improvement, leading to enhanced plaque control and improved patient comfort. Owing to the extent of the gingival enlargement, a segmental surgical approach was adopted to enhance patient comfort, facilitate hemostasis, and allow the close postoperative monitoring of wound healing and plaque control.

Similar successful outcomes following the surgical management of PIGE have been reported in previous case reports. Rao et al. described satisfactory healing following gingivectomy with electrocautery in a medically compromised patient [15]. Similarly, Alfarone et al. [16] and Gupta et al. [17] reported favorable outcomes after external bevel gingivectomy combined with meticulous plaque control and supportive periodontal therapy (SPT). The present case further demonstrates that satisfactory long-term periodontal stability can be achieved despite the continuation of the offending medication, provided that appropriate surgical management is followed by meticulous plaque control and supportive periodontal therapy. No recurrence was observed during the three-year follow-up period.

The absence of clinically evident recurrence during three years of structured supportive periodontal care suggests that sustained plaque control and regular maintenance may have contributed to long-term stability. Since inadequate plaque control and poor adherence to maintenance care increase the likelihood of recurrence, continuous periodontal maintenance is essential for sustained treatment success [18,19].

One limitation of the present case was the increased root exposure and increased clinical crown height following surgical excision, resulting in compromised aesthetics and postoperative dentinal hypersensitivity. However, these are often unavoidable consequences of surgical management in cases of severe generalized gingival enlargement and are outweighed by the restoration of periodontal health, function, and maintainability.

The early recognition of the oral adverse effects of phenytoin requires close collaboration between physicians and dental professionals. Incorporating routine dental examinations into the long-term follow-up of patients receiving phenytoin therapy facilitates timely diagnosis, appropriate periodontal intervention, and the prevention of advanced gingival enlargement. The importance of supportive periodontal therapy should therefore be emphasized by all oral healthcare providers involved in the long-term care of these patients.

## Conclusions

This case demonstrates the successful surgical management of drug-induced gingival enlargement (DIGE) and underscores the importance of early diagnosis and timely intervention in achieving favorable functional and aesthetic outcomes. Greater awareness among both patients and healthcare professionals regarding the oral adverse effects of systemic medications is essential for early recognition and appropriate referral. Close collaboration and effective communication between physicians and oral healthcare providers, together with patient education, meticulous plaque control, and long-term SPT, are critical for achieving favorable clinical outcomes and minimizing the risk of recurrence, thereby improving patients' oral health and quality of life.
